# Longitudinal changes in proportionate mortality due to COVID-19 by occupation in England and Wales

**DOI:** 10.5271/sjweh.4048

**Published:** 2022-10-29

**Authors:** Mark Cherrie, Sarah Rhodes, Jack Wilkinson, William Mueller, Vahe Nafilyan, Martie Van Tongeren, Neil Pearce

**Affiliations:** 1 Institute of Occupational Medicine, Edinburgh, UK.; 2 Centre for Biostatistics, School of Health Sciences, Faculty of Biology, Medicine and Health, School of Health Sciences, The University of Manchester, Manchester, Manchester, UK.; 3 Health Analysis Division, Office for National Statistics, Newport, UK.; 4 Centre for Occupational and Environmental Health, School of Health Sciences, Faculty of Biology, Medicine and Health, University of Manchester, Manchester, Greater Manchester, UK.; 5 Epidemiology and Population Health, London School of Hygiene & Tropical Medicine, London, UK.

**Keywords:** occupational inequality, proportionate mortality analysis, SARS-CoV-2

## Abstract

**Objective:**

This study aimed to understand whether the proportionate mortality of COVID-19 for various occupational groups has varied over the pandemic.

**Methods:**

We used the Office for National Statistics (ONS) mortality data for England and Wales. The deaths (20–64 years) were classified as either COVID-19-related using ICD-10 codes (U07.1, U07.2), or from other causes. Occupational data recorded at the time of death was coded using the SOC10 coding system into 13 groups. Three time periods (TP) were used: (i) January 2020 to September 2020; (ii) October 2020–May 2021; and (iii) June 2021–October 2021. We analyzed the data with logistic regression and compared odds of death by COVID-19 to other causes, adjusting for age, sex, deprivation, region, urban/rural and population density.

**Results:**

Healthcare professionals and associates had a higher proportionate odds of COVID-19 death in TP1 compared to non-essential workers but were not observed to have increased odds thereafter. Medical support staff had increased odds of death from COVID-19 during both TP1 and TP2, but this had reduced by TP3. This latter pattern was also seen for social care, food retail and distribution, and bus and coach drivers. Taxi and cab drivers were the only group that had higher odds of death from COVID-19 compared to other causes throughout the whole period under study [TP1: odds ratio (OR) 2.42, 95% confidence interval (CI) 1.99–2.93; TP2: OR 3.15, 95% CI 2.63–3.78; TP3: OR 1.7, 95% CI 1.26–2.29].

**Conclusion:**

Differences in the odds of death from COVID-19 between occupational groups has declined over the course of the pandemic, although some occupations have remained relatively high throughout.

As of the week ending 4 March 2022, COVID-19 has been involved in 184 327 deaths in the UK, with around 18% occurring in those aged 15–64 years (1). It has been shown that some occupations have had higher rates of death than others. Broadly occupations termed as “essential” (providing crucial public and private services such as healthcare, social care, sanitary services and transportation) have fared worse (2, 3). Specific occupations that have shown elevated risks include healthcare workers (2, 4), taxi/bus drivers (5) and van drivers (2). However, while higher death rates have occurred in certain occupations, studies have found that there is substantial contribution of non-workplace factors (eg, socioeconomic, region, health status), which explain differential risks by occupational group (2, 5).

The reasons for higher risks in certain occupations include both workplace (direct) factors, and (indirect) factors outside of the workplace. Workplace factors include the location of work (indoors or outdoors), the ability to socially distance or avoid contact with suspected COVID-19 cases and the use of personal protective equipment (6). Factors outside of the workplace including housing conditions and occupancy and general deprivation, particularly in those with insecure low-paid jobs. These factors lead certain occupations to have a higher risk of infection, which may in turn lead to higher death rates. People working in these occupations may have higher accumulated SARS-CoV-2 dose, which could lead to higher viral load, which is associated with worse clinical outcomes of COVID-19 disease (7).

Working conditions have fluctuated over the course of the pandemic. Some organizations moved their staff to furlough in the initial stages of the pandemic and then, increasingly, to part- or full-time home-working. Workers also gained more protection to severe illness from COVID-19 from the start of 2021 as several vaccines became available and widely administered. Geographical location also became more important, especially in the second wave where there were localized, tiered restrictions. It is also likely that regions have differing support for non-pharmaceutical interventions (eg, face masks) (8), due to cultural, economic and social factors.

These changes have produced fluctuations in rates of infection, hospitalization and death – over what is commonly referred to as the three waves (spring 2020, winter 2020/2021 and autumn/winter 2021/2022). Previous mortality analyses on English data have been conducted for 2020 only, which covered “wave 1” and some of “wave 2” (2); analyses on Swedish data covered “wave 1” and most of “wave 2” (5), however we are unaware of analyses that have split the mortality data into the different time periods, rather, they have conducted analyses on the combined data (note that time stratified models have been conducted using infections data) (9, 10).

Proportional mortality ratios (PMR) are a measure of relative mortality frequently used in studies assessing occupational risks (11). The proportionate mortality ratio is defined as the proportion of deaths from a particular cause in one occupational group (a) to deaths from other causes (a+c), compared to another occupational group’s proportion of specific deaths (b) to deaths from other causes (b+d), ie, 
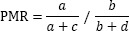
 Meittinen & Wang (12) have shown that the mortality odds ratio (MOR), is a superior measure to the PMR given that it does not require the assumption that the occupational group is not a risk factor for the non-specific causes of death, and is independent of the size of other deaths. Rather than the ratio of the proportions the MOR uses ratios of the odds between occupational groups. Therefore, the MOR is the ratio of deaths attributable to the cause of interest divided by deaths from other causes, compared across occupational groups (ie, 
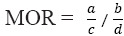
). When the MOR is calculated to be above one for an occupation group of interest, we can conclude there are proportionately more deaths from COVID-19 in that group compared to deaths from other causes, which may suggest that particular occupational exposures lead to increased risk of deaths.

Using logistic regression (as in the current study), the MOR can be estimated after adjustment for key confounders. One advantage of using the MOR is that it did not require denominator data about the total population of the UK. This allowed us to conduct a more up-to-date analysis than can be conducted with analyses which require data linkage or the estimation of population denominators. Also, the MOR is at less risk of bias due to confounding by socioeconomic variables, as deaths due to COVID-19 are compared to deaths from other causes within the same occupational group and this in part adjusts for background differences in the overall mortality risk between occupational groups (13). The method presented differs from the previous occupational COVID-19 mortality studies, which have linked census or administrative and population register-derived data to death records and analyzed time-to-death using cox proportional hazard models (5, 14).

It is also important to determine how proportionate mortality varies across occupational groups *and* time periods over the course of the pandemic. This may point to certain groups of occupations that have (i) had a longstanding higher/lower odds of COVID-19 death, (ii) have recently become at higher odds of COVID-19 death or (iii) have recently become at lower odds of COVID-19 death. Governments can use this as evidence to apply occupation-specific interventions.

We have conducted an analysis of COVID-19 deaths in England and Wales during the period of January 2020 to October 2021. The aim was to understand the changes in deaths related to COVID-19 by occupational groups, over the course of the pandemic, and whether this varied by region of residence.

## Methods

### Deaths data

We used the Office for National Statistics (ONS) death registration data for England and Wales. We included all deaths in the working age population, defined here as aged 20–64 years old. The deaths were categorized as COVID-19 if there was any mention of ICD-10 codes U07.1 (COVID-19, virus identified) or U07.2 (COVID-19, virus not identified) on the death certificate; all other deaths were categorized as not COVID-19 related. We did not include UO9.9 (multisystem inflammatory syndrome associated with COVID-19) or U10.9 (multisystem inflammatory syndrome associated with COVID-19, unspecified), which have only been used since early 2021 (15). The death certificate also contained information on age, sex and postcode.

### Occupational data

Occupational information is recorded at the time of death registration by the informant and coded using the SOC10 (standard occupational classification) coding system (supplementary material, www.sjweh.fi/article/4048). The four-digit SOC10 codes were then grouped into the following categories: healthcare professionals (eg, medical practitioners, pharmacists) and associates (eg, nurses, midwives); medical support staff (eg, ambulance staff, hospital porters); social care; education; food retail and distribution; food production; taxi and cab drivers; bus and coach drivers; van drivers; other transport workers, police and protective services; and sanitary workers (S1). This allowed for comparison with existing studies (3). All other occupations were coded as “non-essential”. There were 33 604 deaths (20%) with only a 2 digit SOC10 code recorded; these were coded as “missing” in the analysis.

### Covariates

We used information on lower super output area (LSOA) – retrieved based on the postcode – of death to gain information on neighborhood deprivation [index of multiple deprivation (IMD) income domain], population density, 8-class urban/rural classification and government office region (GOR). Neighborhood deprivation and population density were z-score standardized (ie, each data point was then interpreted by the number of standard deviations from the sample mean). In the interaction analyses (described below), we coded London, West Midlands and the North-West as binary variables (as there were too few cases to investigate all regions); chosen as they contain the three largest cities – London, Birmingham and Manchester. These three regions had the highest age standardized COVID-19 mortality during wave 1 and wave 2 (16).

### Time periods

We originally pre-specified four time periods (TP) that were related to the extent of community level of infection and the presence of restrictions: January 2020–September 2020 (mixed community infection; mixed restrictions), October 2020–February 2021 (high community infection; high restrictions), March 2021–May 2021 (low community infection; low restrictions), and June 2021–October 2021 (high community infections; low restrictions). However, given low numbers, we merged October 2020–February 2021 and March 2021–May 2021, resulting in October 2020–May 2021. Therefore, we used three TP for the analysis: TP1 (January 2020– September 2020); TP2 (October 2020–May 2021) and TP3 (June 2021–October 2021). These periods therefore contained a mixture of community infection rates which provided sufficient numbers to evaluate occupational differences. These TP roughly correspond to waves 1 (original virus dominant), 2 (Alpha variant dominant), and 3 (Delta variant dominant) in the UK and to the vaccination rollout programme: pre-vaccinated population, increasingly vaccinated population (first dose: 0–60%; second dose: 0–26.7%; booster dose: 0%) and highly vaccinated population (first dose: 68.8–85%; second dose: 45.3–78.1%; third dose: 0–1.8%). Due to low numbers, we further collapsed the new TP to TP2 (October 2020–May 2021) and TP3 (June 2021–October 2021) in the regional analyses.

### Statistical analysis

We conducted a proportionate mortality analysis using logistic regression (17). We adjusted for variables in nested models: firstly adjusted for age, age squared and sex; then additionally for neighborhood deprivation, then region and finally for urban/rural classification and population density (fully adjusted). We then stratified the fully adjusted model by time period. The main results were presented as MOR with 95% confidence intervals (CI).

We then used the fully adjusted model and included a two-way interaction between occupational group and region (with region removed as a covariate), and then stratified the model by TP. We calculated two sets of marginal OR: firstly, the marginal OR and 95% CI for each occupation compared with non-essential workers, for those living in London; secondly, the marginal OR and 95% CI for each occupation compared with non-essential workers, for those not living in London.

A complete case analysis was undertaken. All analyses were conducted in R ver 4.0.2 in the ONS Secure Research Service.

## Results

There were 16 625 deaths (12%) related to COVID-19 from January 2020 to October 2021 in ages 20–64 years old (table 1). Deaths related to COVID-19 peaked between October 2020 and May 2021 (22%); compared to 8% (January 2020–September 2020) and 7% (June 2021–October 2021) (table 2). Deaths were more likely to be among men (62%), and in older age (median 56 years), higher income deprivation (standard median -0.23) and higher population density (standard median -0.20). COVID-19 deaths were concentrated in major conurbations, cities and towns (80%), and in the North West (15%), London (13%) and South East (13%).

**Table 1:** Sample characteristics (N=136 567). Source: Office of National Statistics. [IMD=index of multiple deprivation]

Characteristic	N (%)	Median (Q1–Q3)
COVID-19-related deaths	16 625 (12)	
COVID-19-related deaths by time period		
January 2020–Sept 2020	4756 (8)	
October 2020–May 2021	9298 (22)	
June 2021–October 2021	2571 (7)	
Age (years)		56 (49–61)
Sex (female)	52 343 (38)	
IMD income deprivation ^a^		-0.2275 (-0.8248–0.6394)
Missing	504 (<1)	
Population density ^a^		-0.1959 (-0.7054–0.3432)
Missing	504 (<1)	
Urban rural classification (8-class)		
Major conurbation	47 111 (34)	
Minor conurbation	4965 (4)	
City and town	62 676 (46)	
City and town (sparse setting)	395 (<1)	
Town and fringe	11 317 (8)	
City and town (sparse setting)	768 (<1)	
Town and fringe	11 317 (8)	
Town and fringe (sparse setting)	768 (<1)	
Village and dispersed	7737 (6)	
Village and dispersed (sparse setting)	1094 (<1)	
Missing	504 (<1)	
Government office region		
London	17 360 (13)	
East Midlands	11 400 (8)	
East of England	12 512 (9)	
North East	7683 (6)	
North West	20 591 (15)	
South East	18 377 (13)	
South West	11 520 (8)	
Wales	8226 (6)	
West Midlands	14 681 (11)	
Yorkshire and the Humber	13 713 (10)	
Missing	504 (<1)	

a Z-score scaled.

**Table 2:** COVID-19 deaths in occupational groups, by time period. Source: Office of National Statistics.

Occupational group	Time Period					

	January 2020–September 2020 N (%)		October 2020–May 2021 N (%)		June 2021–October 2021 N (%)	
		
	COVID-19 (N=5403)	Non-COVID-19 (N=54 041)	COVID-19 (N=9298)	Non-COVID-19 (N=32 630)	COVID-19 (N=2731)	Non-COVID-19 (N=32 464)
					
	N (%)	N (%)	N (%)	N (%)	N (%)	N (%)
Non-essential workers	2490 (7.9)	29 084	4052 (20)	16 185	1400 (7.3)	17 751
Healthcare professionals and associates	215 (11.9)	1598	218 (20.4)	848	52 (5)	983
Medical support staff	116 (14.4)	686	101 (24.2)	316	34 (7.8)	402
Social care	372 (10.8)	3082	675 (26.3)	1913	177 (7.7)	2124
Education	141 (7.9)	1639	210 (19.6)	862	68 (6.6)	964
Food retail and distribution	256 (8.9)	2631	521 (26.3)	1457	137 (7.3)	1743
Food production	63 (8.2)	710	100 (19.3)	417	29 (7.0)	387
Taxi and cab drivers	147 (21.7)	530	242 (48.2)	260	54 (13.7)	341
Bus and coach drivers	58 (16.2)	300	104 (40)	156	20 (10)	180
Van drivers	72 (9.6)	678	147 (25.7)	426	31 (6.4)	451
Other transport workers	165 (9.6)	1559	327 (28.2)	829	75 (6.7)	1043
Police and protective services	58 (7.8)	687	92 (19.6)	376	30 (6.9)	406
Sanitary workers	125 (7.9)	1443	204 (20.7)	782	65 (6.6)	911
Missing	478 (4.5)	10061	2305 (22.8)	7803	399 (7.4)	4938

In the fully adjusted models, in comparison to non-essential workers, there was elevated odds of COVID-19 death in all but three of the essential occupational groups (education, police and protective services, and sanitary workers) (table 3). Taxi and cab drivers had the highest relative odds of death from COVID-19 (MOR 2.65; 95% CI 2.37–2.95). Estimates for occupational groups were marginally attenuated after full adjustment, more so among taxi and cab drivers, which was MOR 2.94 (95% CI 2.64–3.28) for the age and sex adjusted model. The other occupations had smaller reductions in MOR, except for food production which substantially increased. We also investigated an expanded group of occupations (S2). There were occupations that were within the non-essential workers group (sales occupations; elementary security occupation; process operatives; managers and directors in retail and wholesale) that had an elevated odds of COVID-19 death compared with corporate managers (S2).

**Table 3:** COVID-19 mortality odds ratio (MOR) for occupational groups. Source: Office of National Statistics. [CI=confidence interval.]

Occupational classification	Adjusted for age and sex	Adjusted for age, sex and deprivation	Adjusted for age, sex, deprivation and region	Adjusted for age, sex, deprivation, region, urban/rural and population density
			
	MOR (95% CI)	MOR (95% CI)	MOR (95% CI)	MOR (95% CI)
Non-essential workers	Ref	Ref	Ref	Ref
Healthcare professionals and associates	1.23 (1.12–1.35)	1.26 (1.14–1.38)	1.26 (1.15–1.39)	1.26 (1.14–1.39)
Medical support staff	1.46 (1.27–1.67)	1.45 (1.26–1.65)	1.46 (1.28–1.67)	1.44 (1.26–1.65)
Social care	1.44 (1.35–1.53)	1.41 (1.32–1.5)	1.42 (1.33–1.52)	1.42 (1.33–1.52)
Education	1.03 (0.93–1.14)	1.06 (0.96–1.18)	1.05 (0.95–1.16)	1.05 (0.95–1.17)
Food retail and distribution	1.3 (1.21–1.4)	1.29 (1.2–1.39)	1.31 (1.22–1.41)	1.31 (1.22–1.41)
Food production	1.01 (0.87–1.16)	1.01 (0.87–1.17)	1.11 (0.96–1.29)	1.24 (1.07–1.43)
Taxi and cab drivers	2.94 (2.64–3.28)	2.87 (2.58–3.2)	2.69 (2.42–3.01)	2.65 (2.37–2.95)
Bus and coach drivers	2.12 (1.81–2.49)	2.07 (1.76–2.43)	2.04 (1.73–2.4)	2.04 (1.73–2.4)
Van drivers	1.26 (1.11–1.43)	1.24 (1.09–1.41)	1.24 (1.09–1.41)	1.23 (1.08–1.4)
Other transport workers	1.22 (1.12–1.33)	1.23 (1.12–1.34)	1.25 (1.14–1.36)	1.26 (1.15–1.37)
Police and protective services	0.89 (0.76–1.04)	0.91 (0.78–1.06)	0.97 (0.83–1.13)	0.98 (0.84–1.14)
Sanitary workers	1.03 (0.93–1.14)	0.98 (0.89–1.09)	0.99 (0.89–1.1)	0.99 (0.89–1.1)
Missing	1.22 (1.16–1.27)	1.17 (1.12–1.22)	1.16 (1.11–1.21)	1.15 (1.1–1.21)

On average the relative difference in the odds of COVID-19 death between the occupational groups and non-essential workers was higher in the second time period than for any other period (table 4). Healthcare professionals and associates had a higher relative odds in TP1 but then were not significantly different to non-essential workers thereafter. Medical support staff had higher odds of death from COVID-19 for longer (TP1 and TP2) but also became not significantly different to non-essential workers by TP3 (although they are still elevated but much less deaths results in a more imprecise estimate). This pattern was also seen for social care, food retail and distribution, and bus and coach drivers. There were two occupations that only had elevated odds in TP2 – van drivers and other transport workers. Taxi and cab drivers were the only group that have had elevated odds of death from COVID-19 at each stage of the pandemic, although this has varied in the magnitude – with TP2 having the highest relative odds of death, then TP1 and then TP3.

**Table 4:** COVID-19 mortality odds ratio (MOR) for occupational groups, by time period. adjusted for age, sex, deprivation, region, urban/rural classification and population density. Source: Office of National Statistics. [CI=confidence interval.]

Occupational classification	Time Period		

	January 2020– September 2020	October 2020–May 2021	June 2021– October 2021
		
	MOR (95% CI)	MOR (95% CI)	MOR (95% CI)
Non-essential workers	Ref	Ref	Ref
Healthcare professionals and associates	1.85 (1.59–2.16)	1.08 (0.92–1.26)	0.84 (0.63–1.12)
Medical Support Staff	2.12 (1.73–2.61)	1.31 (1.04–1.65)	1.21 (0.85–1.73)
Social care	1.6 (1.42–1.8)	1.45 (1.31–1.6)	1.15 (0.97–1.36)
Education	1.17 (0.97–1.4)	1.02 (0.87–1.2)	1.09 (0.84–1.41)
Food retail and distribution	1.23 (1.07–1.41)	1.46 (1.31–1.62)	1.07 (0.89–1.29)
Food production	1.26 (0.97–1.65)	1.12 (0.89–1.4)	1.07 (0.73–1.57)
Taxi and cab drivers	2.42 (1.99–2.93)	3.15 (2.63–3.78)	1.7 (1.26–2.29)
Bus and coach drivers	1.84 (1.37–2.46)	2.44 (1.89–3.15)	1.42 (0.89–2.27)
Van drivers	1.1 (0.86–1.41)	1.33 (1.1–1.62)	0.78 (0.54–1.13)
Other transport workers	1.14 (0.96–1.34)	1.54 (1.35–1.77)	0.9 (0.7–1.14)
Police and protective services	1.03 (0.78–1.36)	1.03 (0.81–1.3)	0.99 (0.68–1.44)
Sanitary workers	1.06 (0.88–1.29)	1 (0.85–1.18)	0.95 (0.73–1.23)
Missing	0.64 (0.58–0.71)	1.26 (1.19–1.34)	0.95 (0.84–1.07)

London was the only region (out of the three tested) that had a significant interaction with occupational group (likelihood ratio test P<0.05); indicating that the odds of COVID-19 death by occupational group was modified by London residence, compared to the rest of England and Wales. The marginal MOR showed that the odds of death from COVID-19 was slightly elevated in London for a number of occupations, and this difference was most apparent during the early pandemic (January 2020–September 2020). The occupational groups that were most reflective of these trends were food production, bus and coach drivers and taxi drivers, although each had small numbers and therefore the estimates have high uncertainty.

## Discussion

### Principal findings

Overall the differences in the odds of death involving COVID-19 between occupational groups have declined over the course of the pandemic. The highest difference between essential and non-essential workers was in the second time period, which might indicate the impact of the Alpha variant, which had higher hospitalization compared to the original virus (18).Low occupational differences by the third time period when the Delta variant was dominant (and also had an increased risk of mortality) (19) suggests that vaccines have had an effect on reducing occupational inequalities in risk of COVID-19 mortality. This is exemplified by healthcare workers who had above average coverage [fully vaccinated (two dose) in 92% of nurses/midwives and 89% of medical practitioners] (20).

Whilst general restrictions were highest in the earlier TP this also corresponded to the time when occupational differences were higher, which suggests that they were not protecting workers who could not work from home. A notable exception is workplace controls in healthcare settings, which were inadequate during the early stages of the pandemic but were enhanced (through adequate provision of PPE and guidance). This may explain the lower odds of COVID-19 death by the later TP (21). However cumulative incidence of COVID-19 mortality, prevalence of naturally acquired immunity though repeated infection and the increasing use of medical treatments to reduce likelihood of death could also have played a role in the findings presented.

An alternative explanation for the patterns observed in the current study is that the odds of deaths involving COVID-19 have increased in the non-essential group, which would attenuate relative differences with the other occupational groups. However, the difference in the odds of deaths involving COVID-19 from TP3–TP1 for the non-essential occupational group was -7.3%. The other occupational groups had differences between -11.6% (police and protective services) and -57.6% (healthcare professionals and associates). This suggests that it is the decline in COVID-19 mortality in ‘essential’ occupations rather than an increase in ‘non-essential’ mortality that is driving the associations observed.

The only occupational group that has seen elevated odds of deaths related to COVID-19 (compared to non-essential occupations) at each stage of the pandemic was taxi and cab drivers. A study using a job exposure matrix assigned taxi drivers as *high risk* for a number of factors associated with increased risk of infection, including: (i) number of adults/adolescents at the same worksite during a typical work day, (ii) indirect contact with adults/adolescents at work within the same workday, (iii) location of work (inside or outside), (iv) elevated risk of contact with adults/adolescents with (suspicion of) COVID-19, (v) social distancing among adults/adolescents at the same work floor (patients, citizen, colleagues), (vi) protection equipment, (vii) migrant workers (proportion of migrant workers), and (viii) low risk for job insecurity (proportion of flexible labor contracts) (6). Taxi drivers have among the smallest indoor working space which is often poorly ventilated; it was found that CO_2_ levels can rise to >2500 ppm and stay high for a working shift (22); this is well above the guidance from the UK’s Health and Safety Executive, which states that action should be taken in any working space with CO_2_ >1500 ppm (23). Changes to the ventilation (eg, air conditioning) within taxis could impact on mortality through reduced exposure to COVID-19 virus specifically, and also air pollution in general (24).Taxi drivers also have lower than average vaccination rates – 83.3% two-dose vaccinated (20).

For those living in London compared to the rest of England and Wales, the odds of deaths involving in COVID-19 were slightly elevated for food production, bus and coach workers, and taxi drivers, compared to non-essential workers, especially in the early stages of the pandemic. Food production was also the only occupation where the MOR substantially increased after adjustment for urban/rural and population density, which provides further support that the likelihood of severe COVID-19 was more geographically patterned in this group. Regional differences may be due to enhancement of occupational risks by individual socioeconomic factors – overcrowding and higher proportion of children in low income families (16). Occupational groups may have enhanced risk of infection and death due to the intensity of the activity – more working hours and more (potential) contact with people with COVID-19. It may also be due to the different mixtures of individual jobs within each job category. For example indoor jobs such as “food, drink and tobacco process operatives” may be over-represented in the food production group in urban regions like London; whereas more outdoor jobs with lower risk such as “fishing and other elementary agricultural occupations n.e.c.” may be in this category in regions like South West – thus skewing the regional differences.

One other UK study has investigated longitudinal differences in excess mortality over the pandemic (25). The analysis was restricted to 2020. The authors found that excess deaths peaked at the end of April 2020 and that essential workers, particularly healthcare workers, were most affected. They found a difference between healthcare professionals/associate professionals and medical support staff, whereby the latter had a longer period of higher excess deaths. Therefore the results on healthcare workers are similar to the current study. They also show that transport and social workers were the most affected groups in November 2020 with approximately 20% excess deaths, in agreement with the current study that showed elevated odds in the first two time periods. There are differences between the studies. They found that food workers, police/protective services and to a lesser extent education workers had higher excess deaths early in the pandemic which then returned back to pre-COVID-19 excess mortality rates. We found that police/protective services and education did not have a higher odds of COVID-19 death compared to essential workers at any time period.

To our knowledge there are no analyses outside of the UK on occupational difference in COVID-19 mortality risks over time. However, there is a study from Norway comparing the infection between the first and the second wave, which roughly correspond to the time periods presented in the current analysis (26). A similar patterns emerged whereby some of the largest reductions in odds of infection were for physicians, nurses, dentists and physiotherapists (ie, healthcare); and largest increases for bartenders, transport conductors and travel stewards (ie, some transport occupations).

**Figure 1:**
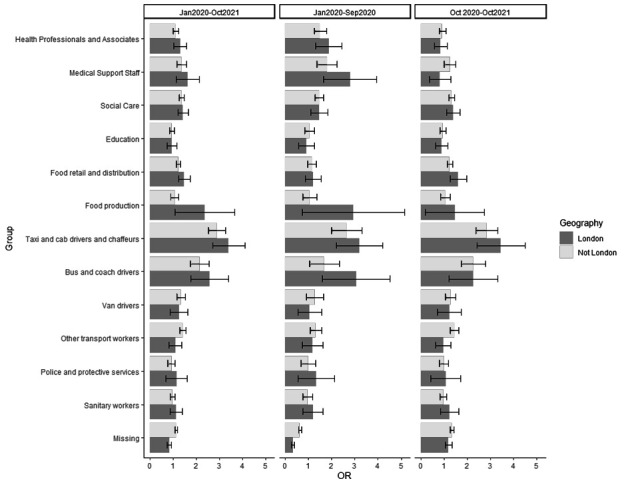
COVID-19 marginal mortality odds ratio (OR) for occupational groups by London/non-London region and time period.

### Strengths

We have conducted a proportionate mortality analysis that covers the hallmarks of a robust analysis (27): a short period of time (which means less bias from population changes), the maximum amount of deaths data available from national registries, all groups have the same access to medical care and diagnosis (due to the UK’s National Health Service) and we account for age at death (through statistical adjustment). We also adjusted for multiple factors identified in directed acyclic graphs in previous analyses (28), including all the variables in the minimal sufficient adjustment set from one study - age, deprivation, geographical region and sex (29).

### Limitations

There is potential misclassification of the deaths for healthcare occupations especially at the beginning of the pandemic, as these individuals were more likely to be tested for COVID-19 and therefore have their death attributed to COVID-19 compared with other occupational groups. In small study samples it is shown that the proportionate mortality ratios may be overestimated (30). Given that the range of the deaths from COVID-19 by occupation was 20–675; overestimation may have been approximately 2.5–22.25% (30). The largest bias would have occurred in the later stages of the pandemic and in the London-stratified models, however these were already predominantly null associations and would therefore be drawn even closer to no effect if corrected. In our regression analysis, we were unable to account for underlying health conditions and ethnicity at the individual level given that it is not recorded on the death certificate and cannot be linked via the individual’s postcode; these have been included in the minimal sufficient adjustment set in two previous papers (2, 3). It is likely that elevated odds for some of the occupations (eg, taxi and cab drivers) where non-white ethnicity is higher would have been attenuated by inclusion of individual ethnicity [due to non-white ethnicity being a risk factor for COVID-19 death (14)]. The risk of death was between two (black African) and five times (Bangladeshi) greater compared to white Britons (31). Given the study design (ie, comparisons made within occupational groups) and the size of the taxi and cab driver effect, we consider it unlikely that both the small sample bias and the inability to account for ethnicity would have affected the main interpretation, that this group had an elevated odds of mortality attributed to COVID-19 across the pandemic.

It was deemed that alternative specifications of variables (ie, including deprivation and population density as a spline, including age as linear rather than quadratic) included in the model would have marginally impacted the results presented, although this has not been formally tested.

### Future work and policy

There are some parts of the study design that could be explored in further work. We have used one definition of a COVID-19 death which includes all deaths “involving” COVID-19. Another definition is deaths “due to” COVID-19, which means that it is the only condition named on the death certificate. This would have reduced the dataset to approximately half the number of deaths (32), which would not have allowed the analysis of a broad set of occupations, time periods and regions. The main analysis focused on groups of occupations, and in doing so masked *within group* variability. It was found that risks of sickness absence due to COVID-19 varied by the specific position within healthcare (33). For the food and drink processing industry, only grain millers were found to have COVID-19 incidence rates (34). It is not just the occupation but also the type of work done in the job that is relevant. Shift work has also been associated with higher risk of severe COVID-19 (35). Further work could also explore changes to the definition of ‘non-essential’ occupations, given that jobs within this category have increased in risk as time has gone on (ie, retail workers). Finally, it is important to triangulate the findings from this study with others eg, the ONS infection survey (36), in order to understand how risk of COVID-19 due to occupational circumstances has changed over time and to make recommendations for policy on added non-pharmaceutical protections in the workplace. The evidence to date indicates that the occupational group with the most severe risk generally has moved from being within healthcare to transport during this pandemic (with the caveat that there were differences between workers within these groups). If this is shown to be the case in multiple countries, then it implies that these workplaces should be prioritized sequentially for the application of the hierarchy of controls in the future (37). Controls could include ventilation systems being tested (and updated if required); face to face contact, hand and surface hygiene policies reviewed; and personal protective equipment stockpiled. The effect on transmission risk from applying these changes should be evidenced by modelling (ie, quantitative microbial assessment models) to provide support for action (38).

### Concluding remarks

Increased odds of COVID-19 death were observed for a number of occupations, such as healthcare professionals and associates and medical support staff compared to non-essential workers, but these differences reduced over the pandemic, with the exception of taxi drivers. Proportionate mortality analysis is a straightforward and practical way to monitor relative differences in COVID-19 mortality by occupation, and their changes over time. It has some considerable practical advantages over other methods since it only requires the mortality data, which is usually readily available and may be less prone to confounding.

## Supplementary material

Supplementary material
