# Supplementary material for: Longitudinal changes in proportionate mortality due to COVID-19 by occupation in England and Wales
Source: Scand J Work Environ Health. 2022 Oct 29;48(8):611–20. doi: 10.5271/sjweh.4048 (PMC10546610; doi:10.5271/sjweh.4048)
Supplement: Supplementary material [file SJWEH-48-611-S001.pdf]

Longitudinal changes in proportionate mortality due to COVID-19 by occupation in England and Wales[1]

by Mark Cherrie, PhD,[2] Sarah Rhodes, MSc, Jack Wilkinson, PhD, William Mueller, MSc, Vahe Nafilyan, PhD, Martie Van Tongeren, PhD, Neil Pearce, PhD

1. Supplementary Material
2. Correspondence to: Mark Cherrie, Institute of Occupational Medicine, Edinburgh, UK. [E-mail: mark.cherrie@iom-world.org]

S1: Occupational coding based on the SOC10 coding system

Group	SOC10 code	SOC Title
Health associate professionals and associates	2211	Medical practitioners
Health associate professionals and associates	2212	Psychologists
Health associate professionals and associates	2213	Pharmacists
Health associate professionals and associates	2231	Nurses
Health associate professionals and associates	2232	Midwives
Health associate professionals and associates	2217	Medical radiographers
Health associate professionals and associates	3213	Paramedics
Health associate professionals and associates	2218	Podiatrists
Health associate professionals and associates	3219	Health associate professionals n.e.c.
Health associate professionals and associates	3217	Pharmaceutical technicians
Health associate professionals and associates	3218	Medical and dental technicians
Health associate professionals and associates	1181	Health services and public health managers and directors
Health associate professionals and associates	1241	Health care practice managers
Health associate professionals and associates	2221	Physiotherapists
Health associate professionals and associates	2219	Health professionals n.e.c.
Health associate professionals and associates	2222	Occupational therapists
Health associate professionals and associates	2223	Speech and language therapists

Health associate professionals and associates	2229	Therapy professionals n.e.c.
Health associate professionals and associates	2214	Ophthalmic opticians
Health associate professionals and associates	2215	Dental practitioners
Health associate professionals and associates	6143	Dental nurses
Support staff	4211	Medical secretaries
Support staff	6141	Nursing auxiliaries and assistants
Support staff	6142	Ambulance staff (excluding paramedics)
Support staff	7114	Pharmacy and other dispensing assistants
Support staff	9271	Hospital porters
Social care	1184	Social services managers and directors
Social care	1242	Residential, day and domiciliary care managers and proprietors
Social care	2442	Social workers
Social care	2444	Clergy
Social care	3231	Youth and community workers
Social care	3234	Housing officers
Social care	2449	Welfare professionals n.e.c.
Social care	3239	Welfare and housing associate professionals n.e.c.
Social care	6144	Houseparents and residential wardens
Social care	6145	Care workers and home carers
Social care	6146	Senior care workers
Social care	6147	Care escorts

Social care	6232	Caretakers
Social care	6148	Undertakers, mortuary and crematorium assistants
Education	231	Teaching and Educational Professionals
Education	2314	Secondary education teaching professionals
Education	2315	Primary and nursery education teaching professionals
Education	2316	Special needs education teaching professionals
Education	6121	Nursery nurses and assistants
Education	6125	Teaching assistants
Food retail and distribution	1254	Shopkeepers and proprietors – wholesale and retail
Food retail and distribution	5431	Butchers
Food retail and distribution	5432	Bakers and flour confectioners
Food retail and distribution	5433	Fishmongers and poultry dressers
Food retail and distribution	7111	Sales and retail assistants
Food retail and distribution	7112	Retail cashiers and check-out operators
Food retail and distribution	9251	Shelf fillers
Food retail and distribution	9259	Elementary sales occupations n.e.c.
Food retail and distribution	1162	Managers and directors in storage and warehousing
Food retail and distribution	1190	Managers and directors in

		retail and wholesale
Food production	8111	Food, drink and tobacco process operatives
Food production	1211	Managers and proprietors in agriculture and horticulture
Food production	1213	Managers and proprietors in forestry, fishing and related services
Food production	5111	Farmers
Food production	5112	Horticultural trades
Food production	5119	Agricultural and fishing trades n.e.c.
Food production	9111	Farm workers
Food production	9119	Fishing and other elementary agriculture occupations n.e.c.
Taxi and cab drivers and chauffeurs	8214	Taxi and cab drivers and chauffeurs
Bus and coach drivers	8213	Bus and coach drivers
Van drivers	8212	Van drivers
Other transport workers	1161	Managers and directors in transport and distribution
Other transport workers	8231	Train and tram drivers
Other transport workers	6215	Rail travel assistants
Other transport workers	8211	Large goods vehicle drivers
Other transport workers	8234	Rail transport operatives
Other transport workers	9211	Postal workers, mail sorters, messengers and couriers
Police and protective services	1172	Senior police officers

Police and protective services	1173	Senior officers in fire, ambulance, prison and related services
Police and protective services	3311	NCOs and other ranks
Police and protective services	3312	Police officers (sergeant and below)
Police and protective services	3313	Fire service officers (watch manager and below)
Police and protective services	3314	Prison service officers (below principal officer)
Police and protective services	3319	Protective service associate professionals n.e.c.
Police and protective services	2443	Probation officers
Sanitary workers	9231	Window cleaners
Sanitary workers	9232	Street cleaners
Sanitary workers	9233	Cleaners and domestics
Sanitary workers	9235	Refuse and salvage occupations
Sanitary workers	9239	Elementary cleaning occupations n.e.c.
Non-essential	All others/not in the list above	All others/not in the list above

S2: Odds of Covid-19 deaths compared with non-essential occupations for expanded occupational grouping

**Occupational classification (SOC 10 code)**	**Unadjusted MOR (95%CI)**	**Adjusted for age and sex (95%CI)**	**Adjusted for age , sex and deprivation (95% CI)**	**Adjusted for age, sex and region (95%CI)**	**Adjusted for age, sex, deprivation, region, urban/rural and population density**
Administrative occupations (41)	0.83 (0.73-0.93)	1.02 (0.9-1.15)	0.99 (0.87-1.11)	0.95 (0.84-1.08)	0.93 (0.82-1.05)
Assemblers and construction operatives (81)	0.79 (0.66-0.94)	0.82 (0.69-0.98)	0.76 (0.64-0.91)	0.79 (0.66-0.94)	0.77 (0.65-0.92)
Business and public service associate professionals (24)	0.79 (0.69-0.91)	0.87 (0.76-1)	0.86 (0.75-0.99)	0.85 (0.74-0.98)	0.84 (0.73-0.97)
Business, media and public service professionals (35)	0.94 (0.81-1.08)	1.01 (0.88-1.17)	1.01 (0.87-1.16)	0.93 (0.8-1.07)	0.91 (0.79-1.06)
Caring personal service occupations (excluding care workers and home carers) (61)	0.8 (0.66-0.95)	1.16 (0.96-1.39)	1.12 (0.93-1.35)	1.06 (0.88-1.28)	1.05 (0.87-1.27)
Culture, media and sports occupations (34)	0.52 (0.43-0.63)	0.58 (0.48-0.7)	0.57 (0.47-0.69)	0.52 (0.43-0.63)	0.51 (0.42-0.62)
Customer service occupations (72)	0.99 (0.81-1.23)	1.28 (1.03-1.58)	1.22 (0.98-1.51)	1.24 (1-1.53)	1.2 (0.97-1.49)
Elementary trades and related occupations (91)	0.75 (0.66-0.86)	0.77 (0.67-0.88)	0.71 (0.62-0.81)	0.74 (0.65-0.85)	0.74 (0.65-0.85)
Health professionals (22)	1.03 (0.9-1.19)	1.33 (1.15-1.54)	1.3 (1.13-1.51)	1.29 (1.11-1.49)	1.27 (1.09-1.46)
Leisure, travel and related personal service occupations (62)	0.91 (0.78-1.06)	1.11 (0.95-1.3)	1.06 (0.9-1.24)	1 (0.85-1.18)	0.98 (0.84-1.16)
Mobile machine and other drivers (82)	0.98 (0.82-1.16)	0.92 (0.77-1.1)	0.86 (0.72-1.03)	0.87 (0.73-1.04)	0.86 (0.72-1.03)
Other managers and proprietors (12)	1.09 (0.96-1.24)	1.15 (1.01-1.3)	1.11 (0.98-1.26)	1.09 (0.96-1.25)	1.1 (0.97-1.25)
Protective service occupations (33)	0.79 (0.65-0.95)	0.76 (0.63-0.93)	0.74 (0.61-0.9)	0.79 (0.65-0.96)	0.79 (0.65-0.96)

Sales occupations (71)	1.01 (0.89-1.14)	1.26 (1.11-1.43)	1.19 (1.05-1.36)	1.19 (1.05-1.36)	1.17 (1.03-1.33)
Science, engineering and technology associate professionals (31)	0.85 (0.68-1.05)	0.9 (0.72-1.11)	0.88 (0.71-1.09)	0.89 (0.72-1.11)	0.87 (0.7-1.08)
Science, research, engineering and technology professionals (21)	0.76 (0.65-0.89)	0.77 (0.66-0.9)	0.77 (0.66-0.9)	0.76 (0.65-0.89)	0.75 (0.64-0.89)
Secretarial and related occupations (42)	0.68 (0.57-0.82)	0.94 (0.78-1.13)	0.92 (0.76-1.11)	0.88 (0.73-1.07)	0.86 (0.71-1.04)
Skilled agricultural and related trades (51)	0.48 (0.39-0.6)	0.47 (0.38-0.58)	0.45 (0.37-0.56)	0.5 (0.4-0.62)	0.53 (0.43-0.66)
Skilled construction and building trades (53)	0.63 (0.55-0.71)	0.59 (0.52-0.67)	0.56 (0.5-0.64)	0.55 (0.49-0.63)	0.55 (0.48-0.62)
Skilled metal, electrical and electronic trades (52)	0.82 (0.73-0.93)	0.77 (0.68-0.87)	0.73 (0.65-0.83)	0.76 (0.67-0.86)	0.75 (0.66-0.85)
Teaching and educational professionals (23)	0.8 (0.68-0.93)	0.96 (0.82-1.13)	0.95 (0.81-1.12)	0.94 (0.8-1.1)	0.92 (0.79-1.09)
Textiles, printing and other (excluding food preparation and hospitality) skilled trades (54)	0.93 (0.76-1.14)	0.94 (0.77-1.15)	0.89 (0.73-1.1)	0.88 (0.72-1.08)	0.87 (0.71-1.07)
Caring personal services (614)	1.23 (1.05-1.45)	1.56 (1.32-1.83)	1.47 (1.25-1.73)	1.47 (1.24-1.74)	1.44 (1.22-1.7)
Elementary administration occupations (921)	1.29 (1.06-1.57)	1.27 (1.04-1.54)	1.21 (0.99-1.47)	1.16 (0.95-1.41)	1.12 (0.92-1.37)
Elementary cleaning occupations (excluding cleaners and domestics) (923)	0.66 (0.52-0.84)	0.65 (0.51-0.84)	0.61 (0.48-0.77)	0.6 (0.47-0.76)	0.59 (0.46-0.75)
Elementary security occupations (924)	1.52 (1.32-1.76)	1.56 (1.35-1.81)	1.45 (1.25-1.68)	1.33 (1.15-1.55)	1.3 (1.12-1.51)
Food preparation and hospitality trades (543)	0.97 (0.84-1.12)	1.07 (0.92-1.23)	1 (0.87-1.16)	0.98 (0.85-1.14)	0.97 (0.84-1.12)
Other elementary occupations (927)	0.84 (0.71-0.99)	1.04 (0.88-1.23)	0.97 (0.82-1.14)	0.96 (0.81-1.13)	0.94 (0.79-1.11)
Plant and machine operatives (812)	0.82 (0.68-0.99)	0.79 (0.65-0.95)	0.74 (0.61-0.89)	0.81 (0.67-0.98)	0.79 (0.65-0.96)

Process operatives (811)	1.37 (1.13-1.65)	1.37 (1.14-1.65)	1.27 (1.05-1.53)	1.35 (1.11-1.63)	1.33 (1.1-1.61)
Bus and coach drivers (8213)	2.01 (1.66-2.44)	1.84 (1.51-2.23)	1.71 (1.41-2.08)	1.66 (1.36-2.01)	1.63 (1.34-1.98)
Care workers and home carers (6145)	1.15 (1.02-1.29)	1.51 (1.33-1.71)	1.41 (1.24-1.6)	1.44 (1.27-1.63)	1.42 (1.25-1.61)
Cleaners and domestics (9233)	0.89 (0.76-1.04)	1.11 (0.95-1.3)	1.02 (0.88-1.2)	1.01 (0.87-1.19)	1 (0.85-1.17)
Elementary storage occupations (9260)	0.9 (0.77-1.06)	0.92 (0.78-1.08)	0.86 (0.73-1.01)	0.88 (0.75-1.04)	0.87 (0.74-1.02)
Large goods vehicle drivers (8211)	1.01 (0.86-1.18)	0.91 (0.78-1.07)	0.88 (0.75-1.03)	0.92 (0.78-1.08)	0.92 (0.79-1.08)
Managers and directors in retail and wholesale (1190)	1.17 (0.95-1.43)	1.34 (1.09-1.65)	1.31 (1.06-1.61)	1.28 (1.04-1.58)	1.27 (1.03-1.56)
Taxi and cab drivers and chauffeurs (8214)	2.87 (2.48-3.32)	2.63 (2.27-3.05)	2.46 (2.12-2.85)	2.25 (1.93-2.61)	2.18 (1.88-2.53)
Van drivers (8212)	1.13 (0.96-1.33)	1.07 (0.91-1.27)	1.01 (0.86-1.2)	1 (0.85-1.19)	0.99 (0.83-1.17)

Source: ONS; n.b. Reference group is Corporate Managers (excluding Retail)
